# Identifying Heat Shock Protein Families from Imbalanced Data by Using Combined Features

**DOI:** 10.1155/2020/8894478

**Published:** 2020-09-23

**Authors:** Xiao-Yang Jing, Feng-Min Li

**Affiliations:** College of Science, Inner Mongolia Agricultural University, Hohhot 010018, China

## Abstract

Heat shock proteins (HSPs) are ubiquitous in living organisms. HSPs are an essential component for cell growth and survival; the main function of HSPs is controlling the folding and unfolding process of proteins. According to molecular function and mass, HSPs are categorized into six different families: HSP20 (small HSPS), HSP40 (J-proteins), HSP60, HSP70, HSP90, and HSP100. In this paper, improved methods for HSP prediction are proposed—the split amino acid composition (SAAC), the dipeptide composition (DC), the conjoint triad feature (CTF), and the pseudoaverage chemical shift (PseACS) were selected to predict the HSPs with a support vector machine (SVM). In order to overcome the imbalance data classification problems, the syntactic minority oversampling technique (SMOTE) was used to balance the dataset. The overall accuracy was 99.72% with a balanced dataset in the jackknife test by using the optimized combination feature SAAC+DC+CTF+PseACS, which was 4.81% higher than the imbalanced dataset with the same combination feature. The Sn, Sp, Acc, and MCC of HSP families in our predictive model were higher than those in existing methods. This improved method may be helpful for protein function prediction.

## 1. Introduction

Heat shock proteins (HSPs) are ubiquitous in living organisms. They act as molecular chaperones by facilitating and maintaining proper protein structure and function [1–4]; in addition, they are involved in various cellular processes such as protein assembly, secretion, transportation, and protein degradation [5, 6]. HSPs are rapidly expressed when the cells are exposed to physiological and environmental conditions such as elevated temperature, infection, and inflammation [7, 8]. Since the HSPs were discovered in 1962 by Ritossa [9], the HSPs have been widely studied, including their involvement in cardiovascular disease, diabetes, cancer [10–14]. According to molecular function and mass, HSPs are categorized into six different families: HSP20 (small HSPS), HSP40 (J-protein), HSP60, HSP70, HSP90, and HSP100 [15]. These families of HSPs have different functions. The HSP20 family is an ATP-independent molecular chaperone. They are efficient in preventing irreversible aggregation processes by binding denatured proteins [16]. The HSP70 family is the most highly conserved among the HSP families; it is an ATP-dependent molecular chaperone that involves protein folding and remodeling [17]. HSP40 is the cochaperone of HSP70, which participates in DNA binding, protein degradation, intracellular signal transduction, exocytosis, endocytosis, viral infection, apoptosis, and heat shock sensing [18]. HSP90 is another ATP-dependent chaperone that controls protein function and activity by facilitating protein folding, binding of ligands to their receptors or targets, or the assembly of multiprotein complexes [19]. The function of the HSP100 protein is to improve the tolerance to temperature and to promote the proteolysis of specific cellular substrates and regulation of transcription [20]. Experimental determination of HSPs are time-consuming and laborious, so it is necessary to use an effective method to predict HSPs. Recently, some computational methods for predicting HSPs have been proposed in the literature. Feng et al. developed a predictor called “iHSP-RAAAC” that selected the reduced amino acid alphabet (RAAA) as a feature vector; the overall predictive accuracy was 87.42% with the jackknife test [21]. Ahmad et al. used the split amino acid composition (SAAC), the dipeptide composition (DC), and PseAAC [22, 23] to identify HSPs; the highest overall predictive accuracy was 90.7% with the jackknife test [24]. Kumar et al. predicted HSPs and non-HSPs, and the best prediction accuracy was 72.98% by using the dipeptide composition (DC) with a 5-fold cross-validation test [25]. Meher et al. used the G-Spaced Amino Acid Pair Composition (GPC) to predict HSPs; a better result was obtained with the jackknife test [26]. Chen et al. summarized the recent advances in machine learning methods for predicting HSPs [27]. Feature selection is generally essential in a classification, and the appropriate integrated feature model generally offers higher accuracy [28]. Hence, the hybrid features have been **s**uccessfully used in recent studies for constructing classifiers [29, 30]. We used the hybrid features to enhance performance. In this paper, the split amino acid composition (SAAC), the dipeptide composition (DC), the conjoint triad feature (CTF), and the pseudoaverage chemical shift (PseACS) were used to predict the HSPs with the same datasets as investigated by Feng et al. Data imbalance is always considered a problem in developing efficient and reliable prediction systems; due to an imbalanced dataset, the classifier would tend towards the majority class. Here, the syntactic minority oversampling technique (SMOTE) was used to solve the problem of imbalance. The overall accuracy was 99.72% with a balanced dataset in the jackknife test by using the optimized combination feature SAAC+DC+CTF+PseACS, which was 4.81% higher than the imbalanced dataset with the same combination feature.

## 2. Material and Methods

### 2.1. Dataset

The benchmark dataset was generated by Feng et al. [21]; the dataset was originally taken from the HSPIR database. In order to reduce homologous bias and redundancy, the program CD-HIT [31] was used to remove those sequences that have ≥40% pairwise sequence identity. 2225 sequences were obtained from different HSP families: the subset *S*_1_ contains 357 sequences, the subset *S*_2_ contains 1279 sequences, the subset *S*_3_ contains 163 sequences, the subset *S*_4_ contains 283 sequences, the subset *S*_5_ contains 58 sequences, and the subset *S*_6_ contains 85 sequences (see Table 1). The dataset can be freely downloaded from http://lin-group.cn/server/iHSP-PseRAAAC. The independent datasets include two datasets: the HGNC dataset and the RICE dataset (see Table 2). The HGNC dataset [32] has 96 human HSPs, and the RICE dataset has 55 RICE HSPs, which obtained 31 HSPs from Wang et al. [33] and 24 HSPs from a single family from Sarkar et al. [34]. The independent dataset can be freely downloaded from http://cabgrid.res.in:8080/ir-hsp.

### 2.2. The Prediction Model Construction Overview

**Figure 1:**
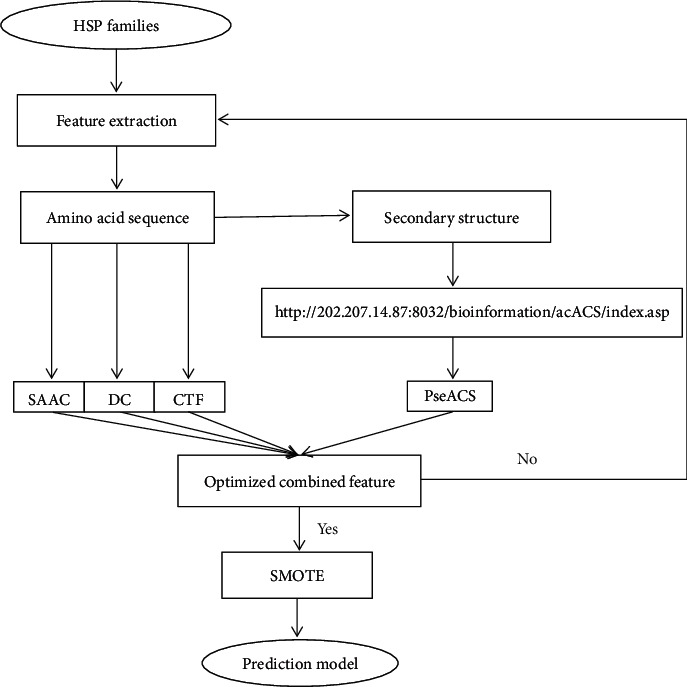
The flowchart of the proposed method. SAAC: split amino acid composition; DC: dipeptide composition; CTF: conjoint triad feature; PseACS: pseudoaverage chemical shift; SMOTE: syntactic minority oversampling technique.

The prediction model process is illustrated in Figure 1. The feature parameters were extracted for the HSPs. By using various information parameters, the prediction results show that better prediction results may be obtained by combining the following four information parameters: the split amino acid composition (SAAC), the dipeptide composition (DC), the conjoint triad feature (CTF), and the pseudoaverage chemical shift (PseACS). In SAAC, the protein sequence was split into the N-terminus segment and the C-terminus segment according to the golden ratio. Among the four feature parameters, the split amino acid composition (SAAC), the dipeptide composition (DC), and the conjoint triad feature (CTF) are based on the protein sequence, while the pseudoaverage chemical shift (PseACS) is related to the protein secondary structure. Therefore, the feature parameters involved both sequence and structure information. The four feature parameters were combined, and the syntactic minority oversampling technique (SMOTE) was used to solve the problem of the imbalance dataset. The overall accuracy (OA) was 99.72% with the balanced dataset, and the result demonstrates that the proposed method is superior to the existing methods.

### 2.3. Feature Extraction Techniques

In order to predict the HSPs, it is very important to choose a classifier and a set of reasonable parameters. In this paper, the split amino acid composition (SAAC), the dipeptide composition (DC) [35], the conjoint triad feature (CTF), and the pseudoaverage chemical shift (PseACS) were used to predict the HSPs.

#### 2.3.1. Split Amino Acid Composition (SAAC)

Split amino acid composition (SAAC) is a feature extraction method based on AAC. In SAAC, the protein sequence is split into various segments; then, the composition of each segment is counted separately [36–39]. It is well known that the golden ratio is ubiquitous in nature. According to the golden ratio, the protein sequence is divided into the N-terminus segment and the C-terminus segment; the ratio of the N-terminus segment to the C-terminus segment is the golden ratio [40]. This method can be represented as follows:

(1) $$\begin{array}{c} \text{SAAC}\_{\text{Gr}}^{1}=\left({\text{AAC}}^{\text{N}},{\text{AAC}}^{\text{C}}\right),\\ {\text{AAC}}^{\text{N}}=\left[x_{1}^{\text{N}},x_{2}^{\text{N}},\cdots ,x_{i}^{\text{N}},\cdots ,x_{20}^{\text{N}}\right],\\ {\text{AAC}}^{\text{C}}=\left[x_{1}^{\text{C}},x_{2}^{\text{C}},\cdots ,x_{i}^{\text{C}},\cdots ,x_{20}^{\text{C}}\right],\\ x_{i}^{\text{N}}=\frac{W_{i}}{L_{\text{N}}},\\ x_{i}^{\text{C}}=\frac{W_{i}}{L_{\text{C}}},\\ \;\left(i=1,2,\cdots ,20\right), \end{array}$$

where Gr^1^ is the 1-step segmentation using the golden ratio, N represents the N-terminus, C represents the C-terminus, *W*_*i*_ is the occurrence of amino acid *i*, *L*_N_ is the length of the N-terminus segment, *L*_C_ is the length of the C-terminus segment.

With this method, we can get SAAC_Gr^2^, SAAC_Gr^3^,….

(2) $$\begin{array}{c} \text{SAAC}\_{\text{Gr}}^{2}=\left({\text{AAC}}_{\text{N}}^{\text{N}},{\text{AAC}}_{\text{N}}^{\text{C}},{\text{AAC}}_{\text{C}}^{\text{N}},{\text{AAC}}_{\text{C}}^{\text{C}}\right),\\ \text{SAAC}\_{\text{Gr}}^{3}=\left({\text{AAC}}_{\text{NN}}^{\text{N}},{\text{AAC}}_{\text{NN}}^{\text{C}},{\text{AAC}}_{\text{NC}}^{\text{N}},{\text{AAC}}_{\text{NC}}^{\text{C}},{\text{AAC}}_{\text{CN}}^{\text{N}},{\text{AAC}}_{\text{CN}}^{\text{C}},{\text{AAC}}_{\text{CC}}^{\text{N}},{\text{AAC}}_{\text{CC}}^{\text{C}}\right). \end{array}$$

#### 2.3.2. Dipeptide Composition (DC)

Dipeptide composition (DC) is a discrete method using sequence neighbor information [27, 41, 42]. The occurrence frequency of each two adjacent amino acid residue was computed; the advantage of DC is that it considers some sequence-order information. It can be calculated as follows:

(3) $$\begin{array}{c} P=\left[f_{1},f_{2},f_{3},\cdots ,f_{i},\cdots ,f_{400}\right],\\ f_{i}=\frac{m_{i}}{L-1}, \end{array}$$

where *m*_*i*_ is the occurrence number of the *i*th dipeptide in the protein sequence, *L* is the length of the protein sequence.

#### 2.3.3. Conjoint Triad Feature (CTF)

The conjoint triad feature (CTF) representation was used by Shen et al. [43]. In this method, the properties of one amino acid and its vicinal amino acids were considered. Three continuous amino acids were regarded as a unit. The 20 amino acids are classified into 7 groups based on dipole moments and the volume of the side chains: {*A*, *G*, *V*}, {*I*, *L*, *F*, *P*}, {*Y*, *M*, *T*, *S*}, {*H*, *N*, *Q*, *W*}, {*R*, *K*}, {*D*, *E*}, and {*C*}. Thus, each protein sequence is represented by a 343- (7 × 7 × 7) dimensional vector, where each element of the vector corresponds to the frequency of the corresponding conjoint triad in the protein sequence. The conjoint triad feature (CTF) has successfully predicted enzyme function [44], protein-protein interactions [45], RNA-protein interactions [46], and nuclear receptors [47]. The features of CTF can be formulated as follows:

(4) $$\begin{array}{c} \text{CTF}=\left[x_{1},x_{2},x_{3},\cdots ,x_{i},\cdots ,x_{343}\right],\\ x_{i}=\frac{n_{i}}{L-2}, \end{array}$$

where *n*_*i*_ is the occurrence number of each triad type of the protein sequence, *L* is the length of the protein sequence.

#### 2.3.4. Pseudoaverage Chemical Shift (PseACS)

Nuclear magnetic resonance (NMR) plays a unique role in studying the structure of proteins because it provides information on the dynamics of the internal motion of proteins on multiple time scales [48]. Protons are sensitive to the chemical environment. The protons in different chemical environments experience slightly different magnetic fields, and they absorb different frequencies in different magnetic fields; the resonant frequencies of the various proteins in relation to a stand are called the chemical shift [49]. As important parameters are measured by nuclear magnetic resonance (NMR) spectroscopy, a chemical shift has been used as a powerful indicator of the protein structure. Several researchers revealed that the averaged chemical shift (ACS) of a particular nucleus in the protein backbone empirically correlates well to its secondary structure [50]. The PseACS web is accessible at http://202.207.14.87:8032/bioinformation/acACS/index.asp.

For a protein *P*, each amino acid in the sequence is substituted by its averaged chemical shift, and *P* can be expressed as follows:

(5) P=A1i,A2i,A3i,⋯,ALi, i=N15,C13α,H1α,H1N,

where ^15^N stands for nitrogen, ^13^C_*α*_ for alpha carbon, ^1^H_*α*_ for alpha hydrogen, and ^1^H_N_ for hydrogen linked with nitrogen.

After, we select *λ* = 54 and *i* = ^15^N, ^13^C_*α*_, ^1^H_*α*_, ^1^H, the PseACS would be expressed as follows:

(6) ϕiλ=1L−λ∑k=1L−λAki−Ak+λi2,i=N15,C13α,H1α,H1N; λ<L,PseACS=ϕi0,ϕi1,ϕi2,⋯,ϕiλ,i=N15,C13α,H1α,H1N.

### 2.4. Syntactic Minority Oversampling Technique (SMOTE)

As shown in Table 1, the numbers of HSP40 are about 4 times, 8 times, 5 times, 22 times, and 15 times that of HSP20, HSP60, HSP70, HSP90, and HSP100, respectively. This leads to imbalance data classification problems. In order to overcome this problem, we used the SMOTE to solve the problem of imbalance. SMOTE is an oversampling approach where the minority class is oversampled by selecting the minority class and creating new synthetic samples along the line segments connecting any or all *K*-Nearest Neighbors which belong to that class [51, 52]. In this paper, the protein numbers of six subfamilies are in equilibrium with SMOTE. This algorithm is implemented by the Weka software. A filter selects SMOTE when the data is loaded, and the parameters adopt the default parameters according to the number of families from small to large; the number of the remaining five families increases in turn to the number of HSP40, which is the largest number of the HSP families. In this way, SMOTE is realized.

### 2.5. Support Vector Machine (SVM)

The support vector machine is a machine learning algorithm, which is based on the statistical learning theory. The basic idea of SVM is to transform the input data into a high-dimensional Hilbert space and then determine the optional separating hyperplane [53, 54]. The radical basis kernel function (RBF) was used to obtain the classification hyperplane with its effectiveness and speed in the training process. To handle a multiclass problem, the regulation parameter *c* and kernel width parameter*γ* were determined via the grid search method. “One-versus-one (OVO)” and “one-versus-rest (OVR)” methods are generally applied to extend the traditional SVM. In this study, the “OVO” strategy was used. The OVO strategy constructs *k* × (*k* − 1)/2 classifiers with each one trained with the data from two different classes. SVM has been successfully applied in the field of computational biology and bioinformatics [55–64]. In this paper, the LibSVM package was used to predict HSPs, which can be downloaded from https://www.csie.ntu.edu.tw/~cjlin/libsvm.

### 2.6. Performance Evaluation

In statistical prediction, three cross-validation tests are commonly used to examine a predictor for its effectiveness in practical application: the *k*-fold cross-validation (subsampling test), the independent dataset test, and the jackknife test. Among the three methods, the jackknife test is deemed the most objective and rigorous one. In the jackknife test, each sample in the training dataset is in turn singled out as an independent test sample and all the rule parameters are calculated based on the remaining dataset without including the one being identified. Hence, the jackknife test was used to evaluate performance in this paper. To evaluate the predictive capability and reliability of our model, the performance of the classification algorithm is measured using the following: sensitivity (Sn), specificity (Sp), accuracy (Acc), Matthew's correlation coefficient (MCC), and overall accuracy (OA) [65–75]. The performance of the classification algorithm is measured through the following:

(7) $$\begin{array}{c} \text{Sn}=\frac{\text{TP}}{\text{TP}+\text{FN}},\\ \text{Sp}=\frac{\text{TN}}{\text{TN}+\text{FP}},\\ \text{MCC}=\frac{\text{TP}\times \text{TN}-\text{FP}\times \text{FN}}{\sqrt{\left(\text{TP}+\text{FP}\right)\times \left(\text{TN}+\text{FN}\right)\times \left(\text{TP}+\text{FN}\right)\times \left(\text{TN}+\text{FP}\right)}},\\ \text{Acc}=\frac{\text{TP}+\text{TN}}{\text{TP}+\text{TN}+\text{FP}+\text{FN}},\\ \text{OA}=\overset{m}{\underset{i=1}{\sum }}{\text{TP}}_{i}/N, \end{array}$$

where TP represents the true positive, TN represents the true negative, FP represents the false positive, and FN represents the false negative. *m* = 6 is the number of subsets, and *N* is the number of total sequences of HSP families.

## 3. Results and Discussion

### 3.1. The Predictive Performance of HSPs

In order to investigate the effectiveness of the predictive model, many characteristic parameters were selected to predict the HSPs [76, 77]. Then, the split amino acid composition (SAAC), the dipeptide composition (DC), the conjoint triad feature (CTF), and the pseudoaverage chemical shift (PseACS) were selected to predict the HSPs.  Table 3 lists the predictive performance of HSPs using individual features with the SVM classification algorithm without SMOTE; the highest overall accuracy (OA) of an individual parameter is 91.38% with the jackknife test by using PseACS. Individual features identify the families of HSPs with an overall accuracy (OA) ranging from 80.92% to 91.38%.

**Figure 2:**
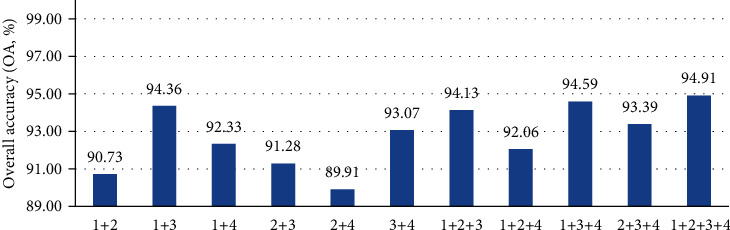
Prediction results of different combined features. Numbers denote features: 1 for DC, 2 for CTF, 3 for PseACS, and 4 for SAAC.

Figure 2 shows the predictive results of different combined features of HSPs with SVM without SMOTE. The results show that the combined feature of SAAC+DC+CTF+PseACS was better than the other parameters. The overall accuracy (OA) of the combined feature of SAAC+DC+CTF+PseACS was 94.91% with the jackknife test. This result indicated that the combined feature was powerful in predicting HSPs.

Table 4 lists the predictive performance of HSP families using the optimized combination feature SAAC+DC+CTF+PseACS with and without SMOTE. In the models with SMOTE, the Sn, Sp, Acc, and MCC of HSP families improved remarkably. For example, for HSP20 with SMOTE, Sn = 100%, Sp = 99.92%, MCC = 1, and Acc = 99.93%, which are 5.65%, 1.34%, 0.08, and 2.04% higher than those without SMOTE. In addition, OA = 99.72% with SMOTE, which is 4.81% higher than HSP families without SMOTE. The results indicate that the combined parameter SAAC+DC+CTF+PseACS with SMOTE was helpful in enhancing predictive performance.

### 3.2. Comparison with Other Algorithms

**Figure 3:**
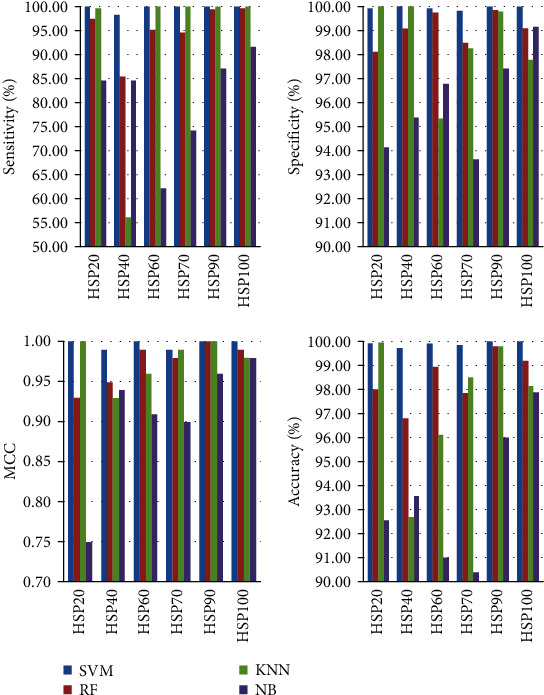
The predictive sensitivity, specificity, MCC, and accuracy of HSPs by using four algorithms.

**Figure 4:**
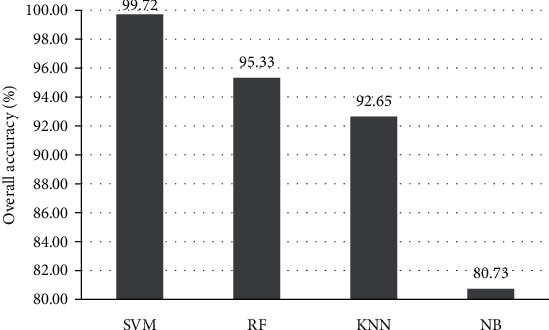
The predictive overall accuracy of HSPs by using four algorithms.

The predictive performance of our predictive model (SVM), Random Forest (RF) [78], Naive Bayes (NB), and *K*-Nearest Neighbors (KNN) [79] is shown in Figures 3 and 4. From Figure 3, we can see that the differences of the Sn, Sp, MCC, and Acc of the HSP families are obvious. The Sn of HSP60, HSP70, HSP90, and HSP100 using SVM and KNN were all 100%. The Sp of HSP20 using KNN and SVM were similar, and the Sp of HSP40 using SVM and KNN were 100%. The MCC of HSP20 and HSP90 using SVM and KNN were both 1. The Acc of HSP20 using KNN and SVM were similar. In addition, from Figure 4, we can see that the value of OA with SVM was 99.72%, which was 4.39%, 7.07%, and 18.99% higher than RF, KNN, and NB, respectively. The highest value of the other parameters was obtained by SVM. Therefore, the experimental results show that SVM has achieved the best measures.

**Figure 5:**
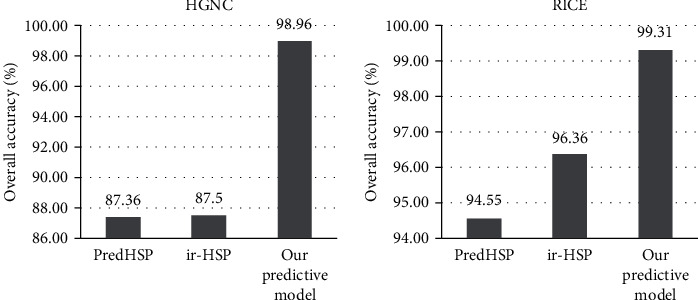
A comparison of the proposed method for independent datasets.

Figure 5 shows the predictive performance of HSP families using independent datasets. In the HGNC independent dataset, the OA of our predictive model was 98.96%, which was 11.60% and 11.46% higher than PredHSP and ir-HSP, respectively. In the RICE independent dataset, the OA of our predictive model reached 99.31%, which was 4.76% and 2.95% higher than PredHSP and ir-HSP, respectively. From the comparison, we can draw a conclusion that the applicability and accuracy of our prediction model for HSP prediction were improved.

### 3.3. Comparison with Existing Methods

In order to evaluate the performance of our predictive model, we made comparisons with existing methods. The method developed by Ahmad et al. did not provide any family-wise accuracy of HSPs, so we compared the effectiveness with iHSP-PseRAAAC, PredHSP, and ir-HSP. The results of the comparisons are shown in Table 5. We can see that the Sn, Sp, Acc, and MCC of HSP families in our predictive model were higher than those of PredHSP, iHSP-PseRAAAC, and ir-HSP. For example, in our predictive model, Sn = 100%, Sp = 99.92%, MCC = 1, and Acc = 99.93% for HSP20 exceeded those of ir-HSP, PredHSP, and iHSP-PseRAAAC. In addition, in our predictive model, Sn = 100 for all HSP families, except for HSP40 Sn = 98.33%. Furthermore, the overall accuracy was 99.72% in our predictive model. These results indicate that our predictive model was superior to existing methods.

## 4. Conclusion

In this work, an optimized classifier for HSP family identification was developed. This model was derived from the SVM machine learning algorithm, and SMOTE was used for the imbalanced data classification problems. The overall accuracy was 99.72% with the balanced dataset and the jackknife test by using the optimized combination feature SAAC+DC+CTF+PseACS. High overall accuracy results indicate that our predictive model is a reliable tool for HSP family prediction. It is known that HSP expression is associated with human diseases, and these families of HSPs have different functions. Therefore, our predictive model will benefit researchers by quickly and effectively identifying HSP families and enabling researchers to design new drugs to achieve the goal of treating diseases.

## Figures and Tables

**Table 1:** The number of sequences in HSP families.

Dataset	Family	Number of HSP samples
*S*_1_	HSP20	357
*S*_2_	HSP40	1279
*S*_3_	HSP60	163
*S*_4_	HSP70	283
*S*_5_	HSP90	58
*S*_6_	HSP100	85
*S*	Overall	2225

**Table 2:** The number of sequences in the independent dataset.

Families	HGNC dataset	RICE dataset	
		Wang et al.	Sarkar et al.
HSP20	11	14	—
HSP40	49	—	—
HSP60	15	4	—
HSP70	17	7	24
HSP90	4	3	—
HSP100	—	3	—
Total	96	31	24

**Table 3:** The predictive results of individual features with the jackknife test by using SVM for HSP families.

Features		HSP families						OA (%)
		HSP20	HSP40	HSP60	HSP70	HSP90	HSP100	
CTF	Sn (%)	74.86	90.92	54.72	67.27	53.85	67.9	80.92
	Sp (%)	95.07	76.19	98.71	96.48	99.86	99.52	
	MCC	0.7	0.68	0.63	0.66	0.69	0.75	
	Acc (%)	91.79	84.68	95.5	92.75	98.76	98.35	
SAAC	Sn (%)	81.07	97.53	58.49	75.9	57.69	74.07	87.25
	Sp (%)	97.7	81.06	99.36	98.26	100	99.48	
	MCC	0.81	0.81	0.7	0.78	0.76	0.78	
	Acc (%)	95	90.55	96.38	95.41	98.99	98.53	
DC	Sn (%)	90.96	96.66	68.55	84.89	63.46	77.78	90.69
	Sp (%)	96.66	90.69	99.11	98.16	100	99.86	
	MCC	0.85	0.88	0.75	0.84	0.79	0.86	
	Acc (%)	95.73	94.13	96.88	96.47	99.13	99.04	
PseACS	Sn (%)	92.37	95.46	75.47	87.41	67.31	83.95	91.38
	Sp (%)	99.01	89.94	98.71	98.16	99.91	99.33	
	MCC	0.92	0.86	0.77	0.86	0.79	0.83	
	Acc (%)	97.94	93.12	97.02	96.79	99.13	98.76	

**Table 4:** The predictive results of HSPs by using the combined feature of SAAC+DC+CTF+PseACS with and without SMOTE.

Features with and without SMOTE (Y/N)			HSP families						OA (%)
			HSP20	HSP40	HSP60	HSP70	HSP90	HSP100	
PseACS+DC+SAAC+CTF	Y	Sn (%)	100	98.33	100	100	100	100	99.72
		Sp (%)	99.92	100	99.92	99.82	100	100	
		MCC	1	0.99	1	0.99	1	1	
		Acc (%)	99.93	99.72	99.93	99.85	100	100	
PseACS+DC+SAAC+CTF	N	Sn (%)	94.35	98.89	81.13	90.29	75	91.36	94.91
		Sp (%)	98.58	94.26	99.6	98.84	100	99.9	
		MCC	0.92	0.94	0.87	0.90	0.86	0.94	
		Acc (%)	97.89	96.93	98.26	97.75	99.4	99.59	

**Table 5:** The comparison of the predictive results between this paper and existing methods.

Method		HSP families					
		HSP20	HSP40	HSP60	HSP70	HSP90	HSP100
iHSP-PseRAAAC^a^	Sn (%)	87.68	95.31	66.87	79.15	51.72	69.41
	Sp (%)	96.36	84.87	98.93	86.54	99.89	99.84
	MCC	0.82	0.99	0.69	0.54	0.3	0.83
	Acc (%)	—	—	—	—	—	—
PredHSP^b^	Sn (%)	92.16	96.09	79.75	91.17	72.41	82.35
	Sp (%)	97.16	86.26	97.24	91.97	99.12	98.08
	MCC	0.87	0.83	0.72	0.71	0.7	0.71
	Acc (%)	96.36	91.91	95.96	91.87	98.43	97.48
ir-HSP^c^	Sn (%)	94.63	97.45	67.92	88.49	75	88.89
	Sp (%)	96.61	95.13	98.86	98.84	99.76	99.57
	MCC	0.8718	0.9276	0.7307	0.8871	0.8112	0.8846
	Acc (%)	96.28	96.47	96.61	97.52	99.17	99.17
Our predictive model	Sn (%)	100	98.33	100	100	100	100
	Sp (%)	99.92	100	99.92	99.82	100	100
	MCC	1	0.99	1	0.99	1	1
	Acc (%)	99.93	99.72	99.93	99.85	100	100

^a^Feng et al. [21]. ^b^Kumar et al. [25]. ^c^Meher et al. [26].

## Data Availability

The data used to support the findings of this study are available from the supplementary materials.

## Supplementary Materials

Supplementary 1 The sequence names of HSP families.

Supplementary 2 The sequence names of the independent datasets.
