# Supplementary material for: Identifying Heat Shock Protein Families from Imbalanced Data by Using Combined Features
Source: Comput Math Methods Med. 2020 Sep 23;2020:8894478. doi: 10.1155/2020/8894478 (PMC7530508; doi:10.1155/2020/8894478)
Supplement: Supplementary 1 — The sequence names of HSP families. [file 8894478.f1.docx]

The dataset contains 2225 heat shock proteins, the number of six families is as follows.

# HSP20(357)

>HSP20_ACMA2 >HSP20_ACMA4 >HSP20_ACLA1 >HSP20_ACFE1 >HSP20_ACFE2 >HSP20_ACFE3

>HSP20_ACCA1 >HSP20_ACCA2 >HSP20_ACCE1 >HSP20_ACCE3 >HSP20_AZVI4 >HSP20_BAAM

>HSP20_BAAN1 >HSP20_BACY2 >HSP20_BAHA1 >HSP20_BAFR >HSP20_BDBA1 >HSP20_BDBA2

>HSP20_BDBA3 >HSP20_BILO1 >HSP20_BOAV >HSP20_BRNA12 >HSP20_BRBR >HSP20_CAHY1

>HSP20_CLBO1 >HSP20_CLKL1 >HSP20_CLTH1 >HSP20_CLTH2 >HSP20_COBU1 >HSP20_DERA1

>HSP20_DERA2 >HSP20_DEAU1 >HSP20_DEAU2 >HSP20_DEAU3 >HSP20_DEAU4 >HSP20_DEAU5

>HSP20_DEDE1 >HSP20_DEMA1 >HSP20_DEMA2 >HSP20_DEMA4 >HSP20_DEVU1 >HSP20_DEVU4

>HSP20_DEVU5 >HSP20_FRTU1 >HSP20_GETH1 >HSP20_GETH2 >HSP20_GETH3 >HSP20_KLPN3

>HSP20_LACA1 >HSP20_LACA2 >HSP20_LADE1 >HSP20_LAFE 1 >HSP20_LEPN1 >HSP20_LEPN3

>HSP20_LECH2 >HSP20_LEME1 >HSP20_MACA1 >HSP20_MAMA1 >HSP20_MELO2 >HSP20_MELO4

>HSP20_MELO5 >HSP20_MEFL1 >HSP20_MENO2 >HSP20_MECA2 >HSP20_MECA3 >HSP20_MECA4

>HSP20_MILU1 >HSP20_MYBO2 >HSP20_MYSM1 >HSP20_MYSM3 >HSP20_MYXA1 >HSP20_MYXA2

>HSP20_NOPU5 >HSP20_PRMI1 >HSP20_PSIN2 >HSP20_PSIN3 >HSP20_RHLE6 >HSP20_RHRU2

>HSP20_RIRI1 >HSP20_STAU1 >HSP20_SYTH1 >HSP20_SYTH2 >HSP20_SYTH3 >HSP20_SYTH4

>HSP20_SYTH5 >HSP20_THTH1 >HSP20_THTH4 >HSP20_THDE2 >HSP20_THDE3 >HSP20_XAOR1

>HSP20_HASA1 >HSP20_HASA3 >HSP20_HAMU1 >HSP20_HAMU2 >HSP20_HAMU5 >HSP20_HAWA1

>HSP20_HAWA2 >HSP20_HAWA3 >HSP20_HAWA4 >HSP20_HALA1 >HSP20_HALA5 >HSP20_IGHO

>HSP20_MESM1 >HSP20_MEBU1 >HSP20_MEBU2 >HSP20_MEAE1 >HSP20_MELA1 >HSP20_MEKA1

>HSP20_MEAC3 >HSP20_MEBA4 >HSP20_MEBA6 >HSP20_MEBA9 >HSP20_MEPA1 >HSP20_MEHU1

>HSP20_METE1 >HSP20_NAEQ1 >HSP20_NIMA1 >HSP20_NIMA2 >HSP20_PYAR2 >HSP20_PYAB1

>HSP20_SUAC1 >HSP20_THAC1 >HSP20_THAC2 >HSP20_THNE3 >HSP20_DIDI1 >HSP20_DIDI2

>HSP20_DIDI3 >HSP20_DIDI4 >HSP20_DIDI5 >HSP20_DIDI7 >HSP20_DIDI9 >HSP20_DIDI12

>HSP20_DIDI15 >HSP20_DIDI16 >HSP20_DIDI20 >HSP20_DIDI21 >HSP20_DIDI22 >HSP20_GLIN2

>HSP20_LEBR1 >HSP20_PLFA2 >HSP20_PLFA3 >HSP20_PLVI2 >HSP20_TRVA1 >HSP20_ASGO4

>HSP20_ASGO5 >HSP20_ASGO6 >HSP20_ASGO7 >HSP20_ASFL3 >HSP20_ASFL4 >HSP20_ASFU1

>HSP20_ASFU2 >HSP20_ASFU4 >HSP20_ASNI5 >HSP20_ASNI7 >HSP20_ASNG3 >HSP20_ASTE1

>HSP20_KLLA1 >HSP20_NECR1 >HSP20_PECH3 >HSP20_PECH4 >HSP20_PECH5 >HSP20_PECH6

>HSP20_PYTR2 >HSP20_SACE1 >HSP20_SACE2 >HSP20_SCJA2 >HSP20_SCJA3 >HSP20_YALI1

>HSP20_YALI2 >HSP20_YALI3 >HSP20_CHRE2 >HSP20_CHRE4 >HSP20_CHRE5 >HSP20_CHRE6

>HSP20_CHRE7 >HSP20_CHRE8 >HSP20_MIPU1 >HSP20_MIPU2 >HSP20_MIPU4 >HSP20_OSLU2

>HSP20_OSLU3 >HSP20_OSTA1 >HSP20_OSTA2 >HSP20_ARTH1 >HSP20_ARTH32 >HSP20_ARTH36

>HSP20_ARTH39 >HSP20_ARTH40 >HSP20_ARTH43 >HSP20_ARTH46 >HSP20_ARTH48 >HSP20_ARTH50

>HSP20_ARTH51 >HSP20_ARTH52 >HSP20_ARTH53 >HSP20_ARTH54 >HSP20_GLMA12 >HSP20_GLMA28

>HSP20_GLMA34 >HSP20_GLMA36 >HSP20_GLMA37 >HSP20_GLMA39 >HSP20_GLMA42 >HSP20_HOVU4

>HSP20_JACU4 >HSP20_METR3 >HSP20_METR4 >HSP20_NITA17 >HSP20_ORSA12 >HSP20_ORSA14

>HSP20_ORSA22 >HSP20_ORSA23 >HSP20_ORSA28 >HSP20_ORSA34 >HSP20_ORSA39 >HSP20_ORSA40

>HSP20_ORSA42 >HSP20_ORSA43 >HSP20_ORSA49 >HSP20_ORSA50 >HSP20_ORSA51 >HSP20_PHPA7

>HSP20_PHPA12 >HSP20_PHPA13 >HSP20_PHPA16 >HSP20_PHPA19 >HSP20_PHPA22 >HSP20_PHPA25

>HSP20_RICO22 >HSP20_RICO23 >HSP20_RICO25 >HSP20_RICO27 >HSP20_RICO29 >HSP20_RICO31

>HSP20_RICO33 >HSP20_RICO35 >HSP20_RICO36 >HSP20_RICO37 >HSP20_RICO39 >HSP20_RICO41

>HSP20_RICO43 >HSP20_RICO45 >HSP20_RICO46 >HSP20_RICO47 >HSP20_RICO48 >HSP20_RICO49

>HSP20_RICO50 >HSP20_SOBI12 >HSP20_SOBI13 >HSP20_SOBI19 >HSP20_SOBI21 >HSP20_SOBI22

>HSP20_SOBI23 >HSP20_SOBI27 >HSP20_SOBI30 >HSP20_SOBI39 >HSP20_SOBI42 >HSP20_SOBI44

>HSP20_VIVI19 >HSP20_VIVI27 >HSP20_VIVI38 >HSP20_VIVI62 >HSP20_VIVI80 >HSP20_VIVI91

>HSP20_VIVI94 >HSP20_VIVI98 >HSP20_VIVI100>HSP20_VIVI102>HSP20_VIVI103>HSP20_VIVI104

>HSP20_VIVI106>HSP20_VIVI108>HSP20_VIVI114>HSP20_VIVI116>HSP20_VIVI117>HSP20_ZEMA16

>HSP20_ZEMA42 >HSP20_ZEMA46 >HSP20_ZEMA51 >HSP20_ZEMA54 >HSP20_ZEMA55 >HSP20_ZEMA56

>HSP20_ZEMA59 >HSP20_AEAE5 >HSP20_AEAE13 >HSP20_AEAE18 >HSP20_BOMO7 >HSP20_BOMO9

>HSP20_BOTA7 >HSP20_BOTA13 >HSP20_BRMA1 >HSP20_BRMA3 >HSP20_BRMA4 >HSP20_BRMA6

>HSP20_BRMA9 >HSP20_BRMA10 >HSP20_BRMA12 >HSP20_BRMA13 >HSP20_BRMA15 >HSP20_CABR2

>HSP20_CABR9 >HSP20_CABR13 >HSP20_CABR14 >HSP20_CABR17 >HSP20_CABR18 >HSP20_CABR19

>HSP20_CABR20 >HSP20_CAEL3 >HSP20_CAEL7 >HSP20_CAEL23 >HSP20_CIIN1 >HSP20_CIIN3

>HSP20_CIIN5 >HSP20_DARE11 >HSP20_DARE12 >HSP20_DARE13 >HSP20_DARE14 >HSP20_DARE18

>HSP20_DARE21 >HSP20_DARE22 >HSP20_DAPU1 >HSP20_DAPU6 >HSP20_DAPU7 >HSP20_DAPU8

>HSP20_DAPU9 >HSP20_DRME5 >HSP20_DRME6 >HSP20_DRME7 >HSP20_DRME8 >HSP20_DRME9

>HSP20_DRME10 >HSP20_DRME11 >HSP20_DRME12 >HSP20_GAGA6 >HSP20_GAGA10 >HSP20_GAGA11

>HSP20_HOSA1 >HSP20_HOSA12 >HSP20_HOSA23 >HSP20_MODO2 >HSP20_MODO5 >HSP20_MUMU15

>HSP20_PATR10 >HSP20_RANO11 >HSP20_SCJA7 >HSP20_SCJA9 >HSP20_SCJA20 >HSP20_SCJA21

>HSP20_SCJA24 >HSP20_TASO1 >HSP20_TENI4 >HSP20_TENI9 >HSP20_TRCA5 >HSP20_TRCA9

>HSP20_TRCA11 >HSP20_XETR7 >HSP20_XETR13

# HSP40(1279)

>HSP40_ACMA4 >HSP40_ACMA6 >HSP40_ACMA8 >HSP40_ACMA10 >HSP40_ACMA11 >HSP40_ACMA12

>HSP40_ACCA2 >HSP40_ACCA3 >HSP40_ACBA2 >HSP40_ACBA3 >HSP40_ACBA4 >HSP40_ACBA5

>HSP40_ACSU2 >HSP40_ACSU3 >HSP40_AEHY3 >HSP40_AEHY5 >HSP40_AGRA6 >HSP40_AGTU2

>HSP40_AGTU4 >HSP40_AGTU6 >HSP40_AGVI2 >HSP40_ANVA2 >HSP40_ANVA6 >HSP40_ANVA8

>HSP40_ANVA9 >HSP40_ANVA10 >HSP40_ANVA13 >HSP40_BAFR1 >HSP40_BDBA2 >HSP40_BDBA3

>HSP40_BDBA4 >HSP40_BEIN4 >HSP40_BEIN5 >HSP40_BIAN2 >HSP40_BOBU1 >HSP40_BOBU2

>HSP40_BOBU3 >HSP40_BRBR2 >HSP40_BRBR3 >HSP40_BRBR4 >HSP40_BRAB2 >HSP40_CAFE3

>HSP40_CAHO3 >HSP40_CAJE1 >HSP40_CAJE2 >HSP40_CAJE3 >HSP40_CAJE4 >HSP40_CACR3

>HSP40_CACR4 >HSP40_CACR5 >HSP40_CACR6 >HSP40_CLCE2 >HSP40_CLKL3 >HSP40_CLKL4

>HSP40_CLPE2 >HSP40_DERA1 >HSP40_DEAU3 >HSP40_DEAU4 >HSP40_DEAU5 >HSP40_DEAU6

>HSP40_DEDE3 >HSP40_DEMA2 >HSP40_DEMA3 >HSP40_DEMA4 >HSP40_DEMA6 >HSP40_DEMA7

>HSP40_ENFA2 >HSP40_FRTU2 >HSP40_FUNU2 >HSP40_HEPY3 >HSP40_LACR2 >HSP40_LEPN3

>HSP40_LEPN4 >HSP40_LECH2 >HSP40_LECH3 >HSP40_LECH5 >HSP40_LEME1 >HSP40_MAMA3

>HSP40_MAMA5 >HSP40_MAMA6 >HSP40_MELO4 >HSP40_MELO6 >HSP40_MELO7 >HSP40_MEFL2

>HSP40_MERA5 >HSP40_MECA4 >HSP40_MILU2 >HSP40_MILU4 >HSP40_MIAE2 >HSP40_MIAE4

>HSP40_MIAE7 >HSP40_MIAE9 >HSP40_MIAE10 >HSP40_MIAE11 >HSP40_MIAE12 >HSP40_MIAE13

>HSP40_MYAV3 >HSP40_MYUL1 >HSP40_MYCO1 >HSP40_MYGE2 >HSP40_MYGE3 >HSP40_MYMY1

>HSP40_MYPN1 >HSP40_MYPN2 >HSP40_MYXA2 >HSP40_MYXA3 >HSP40_MYXA4 >HSP40_MYXA5

>HSP40_MYXA6 >HSP40_MYXA7 >HSP40_MYXA8 >HSP40_MYXA9 >HSP40_MYXA10 >HSP40_MYXA11

>HSP40_MYXA12 >HSP40_MYXA13 >HSP40_MYXA14 >HSP40_MYXA15 >HSP40_MYXA16 >HSP40_NEGO1

>HSP40_NEME3 >HSP40_NIWI3 >HSP40_NIWI4 >HSP40_NIEU3 >HSP40_NOPU9 >HSP40_NOPU10

>HSP40_NOPU13 >HSP40_PHPR4 >HSP40_PHPR5 >HSP40_PHPR6 >HSP40_PRAC2 >HSP40_PRMI3

>HSP40_PSAE4 >HSP40_PSFL3 >HSP40_PSFL7 >HSP40_PSPU3 >HSP40_PSIN3 >HSP40_PSIN6

>HSP40_PSIN7 >HSP40_RHLE3 >HSP40_RHSP2 >HSP40_RHRU2 >HSP40_RHRU4 >HSP40_RHRU5

>HSP40_RHRU7 >HSP40_RIRI2 >HSP40_SATP5 >HSP40_SHBO4 >HSP40_THTH2 >HSP40_THDE3

>HSP40_TRPA1 >HSP40_TRPA2 >HSP40_VIHA5 >HSP40_XAAU3 >HSP40_XAOR2 >HSP40_XAOR4

>HSP40_YEPE3 >HSP40_ZYMO3 >HSP40_ZYMO4 >HSP40_ZYMO5 >HSP40_HASA3 >HSP40_HASA4

>HSP40_HAMU2 >HSP40_HAMU3 >HSP40_HAMU5 >HSP40_HAWA3 >HSP40_HAWA4 >HSP40_HALA2

>HSP40_HALA3 >HSP40_MEBU2 >HSP40_MELA2 >HSP40_MEAC2 >HSP40_MEMA1 >HSP40_MEHU2

>HSP40_MEHU3 >HSP40_MEHU4 >HSP40_NIMA2 >HSP40_NIMA3 >HSP40_NIMA4 >HSP40_THVO1

>HSP40_CRHO5 >HSP40_CRHO6 >HSP40_CRHO9 >HSP40_CRHO10 >HSP40_CRHO11 >HSP40_CRHO17

>HSP40_CRHO19 >HSP40_CRHO22 >HSP40_CRHO23 >HSP40_CRHO24 >HSP40_CRHO25 >HSP40_CRPR1

>HSP40_CRPR2 >HSP40_CRPR5 >HSP40_CRPR8 >HSP40_CRPR11 >HSP40_CRPR12 >HSP40_CRPR15

>HSP40_CRPR16 >HSP40_CRPR17 >HSP40_CRPR18 >HSP40_CRPR20 >HSP40_CRPR22 >HSP40_DIDI1

>HSP40_DIDI3 >HSP40_DIDI4 >HSP40_DIDI8 >HSP40_DIDI9 >HSP40_DIDI13 >HSP40_DIDI14

>HSP40_DIDI15 >HSP40_DIDI16 >HSP40_DIDI17 >HSP40_DIDI18 >HSP40_DIDI20 >HSP40_DIDI21

>HSP40_DIDI22 >HSP40_DIDI24 >HSP40_DIDI26 >HSP40_DIDI27 >HSP40_DIDI28 >HSP40_DIDI29

>HSP40_DIFA3 >HSP40_DIFA4 >HSP40_DIFA5 >HSP40_DIFA6 >HSP40_DIFA7 >HSP40_DIFA8

>HSP40_DIFA12 >HSP40_DIFA14 >HSP40_DIFA15 >HSP40_DIFA16 >HSP40_DIFA18 >HSP40_DIFA20

>HSP40_DIFA21 >HSP40_DIFA22 >HSP40_DIFA24 >HSP40_DIFA25 >HSP40_DIFA27 >HSP40_ENHI5

>HSP40_ENHI6 >HSP40_ENHI7 >HSP40_ENHI9 >HSP40_ENHI10 >HSP40_ENHI11 >HSP40_ENHI12

>HSP40_ENHI13 >HSP40_ENHI14 >HSP40_ENHI15 >HSP40_ENHI16 >HSP40_ENHI17 >HSP40_ENHI18

>HSP40_ENHI19 >HSP40_ENHI21 >HSP40_GIIN1 >HSP40_GIIN2 >HSP40_GIIN3 >HSP40_GIIN4

>HSP40_GIIN5 >HSP40_GIIN6 >HSP40_GIIN7 >HSP40_GIIN8 >HSP40_LEBR1 >HSP40_LEBR2

>HSP40_LEBR4 >HSP40_LEBR5 >HSP40_LEBR7 >HSP40_LEBR8 >HSP40_LEBR11 >HSP40_LEBR12

>HSP40_LEBR13 >HSP40_LEBR14 >HSP40_LEBR16 >HSP40_LEBR17 >HSP40_LEBR19 >HSP40_LEBR20

>HSP40_LEBR21 >HSP40_LEBR22 >HSP40_LEBR23 >HSP40_LEBR24 >HSP40_LEBR26 >HSP40_LEBR27

>HSP40_LEBR28 >HSP40_LEBR29 >HSP40_LEBR30 >HSP40_LEBR31 >HSP40_LEBR32 >HSP40_LEBR33

>HSP40_LEBR34 >HSP40_LEBR36 >HSP40_LEBR37 >HSP40_LEBR38 >HSP40_LEBR39 >HSP40_LEBR40

>HSP40_LEBR41 >HSP40_LEBR42 >HSP40_LEBR43 >HSP40_LEBR44 >HSP40_LEBR46 >HSP40_LEBR47

>HSP40_LEBR48 >HSP40_LEBR49 >HSP40_LEBR50 >HSP40_LEBR51 >HSP40_LEBR52 >HSP40_LEBR53

>HSP40_LEBR54 >HSP40_LEBR55 >HSP40_LEBR56 >HSP40_LEBR57 >HSP40_LEBR59 >HSP40_LEBR61

>HSP40_LEBR62 >HSP40_LEBR63 >HSP40_LEBR64 >HSP40_LEBR65 >HSP40_LEBR66 >HSP40_LEBR67

>HSP40_LEBR68 >HSP40_LEBR69 >HSP40_LEBR70 >HSP40_LEBR71 >HSP40_LEBR72 >HSP40_PHIN1

>HSP40_PHIN4 >HSP40_PHIN7 >HSP40_PHIN8 >HSP40_PHIN9 >HSP40_PHIN10 >HSP40_PHIN11

>HSP40_PHIN12 >HSP40_PHIN13 >HSP40_PHIN14 >HSP40_PHIN16 >HSP40_PHIN17 >HSP40_PHIN18

>HSP40_PHIN19 >HSP40_PHIN20 >HSP40_PHIN21 >HSP40_PHIN22 >HSP40_PHIN23 >HSP40_PHIN24

>HSP40_PHIN25 >HSP40_PHIN27 >HSP40_PHIN28 >HSP40_PHIN29 >HSP40_PHIN30 >HSP40_PHIN31

>HSP40_PHIN32 >HSP40_PHIN33 >HSP40_PHIN35 >HSP40_PHIN36 >HSP40_PHIN37 >HSP40_PHIN38

>HSP40_PHIN39 >HSP40_PHIN40 >HSP40_PHIN41 >HSP40_PHIN42 >HSP40_PHIN43 >HSP40_PLFA1

>HSP40_PLFA4 >HSP40_PLFA5 >HSP40_PLFA7 >HSP40_PLFA8 >HSP40_PLFA9 >HSP40_PLFA10

>HSP40_PLFA12 >HSP40_PLFA13 >HSP40_PLFA14 >HSP40_PLFA15 >HSP40_PLFA16 >HSP40_PLFA17

>HSP40_PLFA18 >HSP40_PLFA19 >HSP40_PLFA20 >HSP40_PLFA22 >HSP40_PLFA24 >HSP40_PLFA28

>HSP40_PLFA31 >HSP40_PLFA32 >HSP40_PLFA33 >HSP40_PLFA36 >HSP40_PLFA38 >HSP40_PLFA39

>HSP40_PLFA40 >HSP40_PLFA41 >HSP40_PLFA42 >HSP40_PLFA44 >HSP40_PLVI5 >HSP40_PLVI8

>HSP40_PLVI9 >HSP40_PLVI11 >HSP40_PLVI13 >HSP40_PLVI14 >HSP40_PLVI15 >HSP40_PLVI16

>HSP40_PLVI19 >HSP40_PLVI20 >HSP40_PLVI21 >HSP40_PLVI22 >HSP40_PLVI24 >HSP40_PLVI26

>HSP40_PLVI28 >HSP40_PLVI29 >HSP40_PLVI30 >HSP40_TETH1 >HSP40_TETH3 >HSP40_TETH5

>HSP40_TETH6 >HSP40_TETH7 >HSP40_TETH8 >HSP40_TETH9 >HSP40_TETH10 >HSP40_TETH11

>HSP40_TETH12 >HSP40_TETH13 >HSP40_TETH14 >HSP40_TETH15 >HSP40_TETH16 >HSP40_TETH17

>HSP40_TETH18 >HSP40_TETH19 >HSP40_TETH20 >HSP40_TETH21 >HSP40_TETH22 >HSP40_TETH23

>HSP40_TETH24 >HSP40_TETH25 >HSP40_TETH26 >HSP40_TETH27 >HSP40_TRVA1 >HSP40_TRVA2

>HSP40_TRVA3 >HSP40_TRVA4 >HSP40_TRVA5 >HSP40_TRVA6 >HSP40_TRVA10 >HSP40_TRVA11

>HSP40_TRVA13 >HSP40_TRVA16 >HSP40_TRVA18 >HSP40_TRVA19 >HSP40_TRVA20 >HSP40_TRVA22

>HSP40_TRVA23 >HSP40_TRVA24 >HSP40_TRVA25 >HSP40_TRVA26 >HSP40_TRVA27 >HSP40_TRVA29

>HSP40_TRVA30 >HSP40_TRVA31 >HSP40_TRVA32 >HSP40_TRVA33 >HSP40_TRVA34 >HSP40_TRVA35

>HSP40_TRVA36 >HSP40_TRVA38 >HSP40_TRVA39 >HSP40_TRVA40 >HSP40_TRVA41 >HSP40_TRVA42

>HSP40_TRVA43 >HSP40_ASGO4 >HSP40_ASGO6 >HSP40_ASGO8 >HSP40_ASGO9 >HSP40_ASGO10

>HSP40_ASGO12 >HSP40_ASGO15 >HSP40_ASGO16 >HSP40_ASGO18 >HSP40_ASGO19 >HSP40_ASCL5

>HSP40_ASCL8 >HSP40_ASCL9 >HSP40_ASCL16 >HSP40_ASFL16 >HSP40_ASFU1 >HSP40_ASFU4

>HSP40_ASFU7 >HSP40_ASFU11 >HSP40_ASFU16 >HSP40_ASFU17 >HSP40_ASFU19 >HSP40_ASFU20

>HSP40_ASFU21 >HSP40_ASFU23 >HSP40_ASNI7 >HSP40_ASNI8 >HSP40_ASNI12 >HSP40_ASNI18

>HSP40_ASNI23 >HSP40_ASNI25 >HSP40_ASNG10 >HSP40_ASNG11 >HSP40_ASNG14 >HSP40_ASNG15

>HSP40_ASNG19 >HSP40_ASNG20 >HSP40_ASOR15 >HSP40_ASOR23 >HSP40_ASTE6 >HSP40_ASTE10

>HSP40_ASTE12 >HSP40_ASTE26 >HSP40_CAAL3 >HSP40_CAAL11 >HSP40_CAAL15 >HSP40_CAAL20

>HSP40_CAAL25 >HSP40_CATR1 >HSP40_CATR2 >HSP40_CATR7 >HSP40_CATR12 >HSP40_CATR15

>HSP40_CATR17 >HSP40_CATR20 >HSP40_CATR23 >HSP40_CRNE1 >HSP40_CRNE4 >HSP40_CRNE6

>HSP40_CRNE8 >HSP40_CRNE9 >HSP40_CRNE10 >HSP40_CRNE11 >HSP40_CRNE13 >HSP40_CRNE14

>HSP40_CRNE15 >HSP40_CRNE16 >HSP40_CRNE17 >HSP40_CRNE18 >HSP40_CRNE19 >HSP40_CRNE20

>HSP40_CRNE21 >HSP40_CRNE22 >HSP40_CRNE23 >HSP40_CRNE24 >HSP40_DEHA3 >HSP40_DEHA9

>HSP40_DEHA10 >HSP40_DEHA13 >HSP40_DEHA15 >HSP40_DEHA16 >HSP40_DEHA19 >HSP40_DEHA21

>HSP40_DEHA22 >HSP40_DEHA24 >HSP40_KLLA5 >HSP40_KLLA8 >HSP40_KLLA9 >HSP40_KLLA10

>HSP40_KLLA15 >HSP40_KLLA16 >HSP40_KLLA18 >HSP40_KLLA20 >HSP40_MICA12 >HSP40_MICA14

>HSP40_MICA15 >HSP40_MICA17 >HSP40_MICA20 >HSP40_MICA25 >HSP40_MICA26 >HSP40_NEFI7

>HSP40_NECR7 >HSP40_NECR8 >HSP40_NECR9 >HSP40_NECR10 >HSP40_NECR11 >HSP40_NECR12

>HSP40_NECR13 >HSP40_NECR14 >HSP40_NECR17 >HSP40_NECR18 >HSP40_NECR19 >HSP40_NECR20

>HSP40_NECR21 >HSP40_NECR23 >HSP40_NECR24 >HSP40_PECH19 >HSP40_PECH21 >HSP40_PIPA1

>HSP40_PIPA6 >HSP40_PIPA7 >HSP40_PIPA8 >HSP40_PIPA11 >HSP40_PIPA13 >HSP40_PIPA14

>HSP40_PIPA15 >HSP40_PIPA16 >HSP40_PIPA17 >HSP40_PIPA18 >HSP40_PIPA20 >HSP40_PIPA22

>HSP40_PIST11 >HSP40_PIST12 >HSP40_PIST13 >HSP40_PIST22 >HSP40_PYTR6 >HSP40_PYTR8

>HSP40_PYTR9 >HSP40_PYTR10 >HSP40_PYTR13 >HSP40_PYTR15 >HSP40_PYTR17 >HSP40_PYTR18

>HSP40_PYTR20 >HSP40_PYTR21 >HSP40_PYTR22 >HSP40_PYTR23 >HSP40_PYTR24 >HSP40_PYTR25

>HSP40_PYTR26 >HSP40_PYTR27 >HSP40_PYTR29 >HSP40_PYTR30 >HSP40_PYTR31 >HSP40_SACE1

>HSP40_SACE3 >HSP40_SACE4 >HSP40_SACE7 >HSP40_SACE11 >HSP40_SACE14 >HSP40_SACE16

>HSP40_SACE18 >HSP40_SACE20 >HSP40_SACR6 >HSP40_SACR17 >HSP40_SCJA4 >HSP40_SCJA6

>HSP40_SCJA7 >HSP40_SCJA8 >HSP40_SCJA9 >HSP40_SCJA10 >HSP40_SCJA13 >HSP40_SCJA14

>HSP40_SCJA16 >HSP40_SCJA17 >HSP40_SCJA19 >HSP40_SCJA20 >HSP40_SCJA22 >HSP40_SCJA23

>HSP40_SCJA24 >HSP40_SCPO1 >HSP40_SCPO8 >HSP40_SCPO11 >HSP40_SCPO12 >HSP40_SCPO13

>HSP40_SCPO14 >HSP40_SCPO15 >HSP40_SCPO16 >HSP40_SCPO18 >HSP40_SCPO20 >HSP40_SCPO21

>HSP40_SCPO23 >HSP40_SCPO25 >HSP40_YALI1 >HSP40_YALI6 >HSP40_YALI7 >HSP40_YALI9

>HSP40_YALI10 >HSP40_YALI11 >HSP40_YALI13 >HSP40_YALI15 >HSP40_YALI16 >HSP40_YALI17

>HSP40_YALI18 >HSP40_YALI19 >HSP40_YALI21 >HSP40_YALI22 >HSP40_YALI23 >HSP40_YALI24

>HSP40_CHRE3 >HSP40_CHRE8 >HSP40_CHRE9 >HSP40_CHRE10 >HSP40_CHRE11 >HSP40_CHRE12

>HSP40_CHRE13 >HSP40_CHRE14 >HSP40_CHRE15 >HSP40_CHRE16 >HSP40_CHRE17 >HSP40_CHRE18

>HSP40_CHRE19 >HSP40_CHRE20 >HSP40_CHRE21 >HSP40_CHRE22 >HSP40_CHRE24 >HSP40_CHRE25

>HSP40_CHRE26 >HSP40_CHRE27 >HSP40_CHRE28 >HSP40_CHRE31 >HSP40_CHRE32 >HSP40_CHRE33

>HSP40_CHRE34 >HSP40_CHRE35 >HSP40_CHRE36 >HSP40_CHRE37 >HSP40_CHRE38 >HSP40_CHRE40

>HSP40_CHRE41 >HSP40_CHRE42 >HSP40_CHRE43 >HSP40_CHRE44 >HSP40_CHRE45 >HSP40_CHRE46

>HSP40_CHRE48 >HSP40_CHRE49 >HSP40_CHRE50 >HSP40_CHRE51 >HSP40_CHRE53 >HSP40_CHRE54

>HSP40_CHRE56 >HSP40_CHRE57 >HSP40_CHRE58 >HSP40_CHRE59 >HSP40_CHRE60 >HSP40_MIPU5

>HSP40_MIPU6 >HSP40_MIPU8 >HSP40_MIPU9 >HSP40_MIPU10 >HSP40_MIPU11 >HSP40_MIPU12

>HSP40_MIPU14 >HSP40_MIPU17 >HSP40_MIPU19 >HSP40_MIPU20 >HSP40_MIPU21 >HSP40_MIPU25

>HSP40_MIPU26 >HSP40_MIPU27 >HSP40_MIPU28 >HSP40_MIPU29 >HSP40_MIPU30 >HSP40_MIPU31

>HSP40_MIPU32 >HSP40_MIPU33 >HSP40_MIPU35 >HSP40_MIPU38 >HSP40_MIPU39 >HSP40_MIPU40

>HSP40_MIPU41 >HSP40_MIPU42 >HSP40_MIPU45 >HSP40_MIPU46 >HSP40_MIPU47 >HSP40_MIPU48

>HSP40_MIPU49 >HSP40_MIPU52 >HSP40_MIPU53 >HSP40_MIPU54 >HSP40_MIPU56 >HSP40_MIPU57

>HSP40_MIPU58 >HSP40_MIPU59 >HSP40_MIPU61 >HSP40_MIPU62 >HSP40_MIPU63 >HSP40_MIPU64

>HSP40_MIPU65 >HSP40_MIPU66 >HSP40_OSLU4 >HSP40_OSLU6 >HSP40_OSLU7 >HSP40_OSLU12

>HSP40_OSLU15 >HSP40_OSLU16 >HSP40_OSLU17 >HSP40_OSLU18 >HSP40_OSLU24 >HSP40_OSLU25

>HSP40_OSLU26 >HSP40_OSLU27 >HSP40_OSLU28 >HSP40_OSLU29 >HSP40_OSLU30 >HSP40_OSLU35

>HSP40_OSLU46 >HSP40_OSLU47 >HSP40_OSLU51 >HSP40_OSLU52 >HSP40_OSLU53 >HSP40_OSLU54

>HSP40_OSLU55 >HSP40_OSLU57 >HSP40_OSTA3 >HSP40_OSTA5 >HSP40_OSTA6 >HSP40_OSTA7

>HSP40_OSTA8 >HSP40_OSTA9 >HSP40_OSTA10 >HSP40_OSTA11 >HSP40_OSTA12 >HSP40_OSTA14

>HSP40_OSTA15 >HSP40_OSTA16 >HSP40_OSTA17 >HSP40_OSTA18 >HSP40_OSTA19 >HSP40_OSTA20

>HSP40_OSTA22 >HSP40_OSTA23 >HSP40_OSTA24 >HSP40_OSTA25 >HSP40_OSTA26 >HSP40_OSTA27

>HSP40_OSTA28 >HSP40_OSTA29 >HSP40_OSTA30 >HSP40_OSTA31 >HSP40_OSTA33 >HSP40_OSTA35

>HSP40_OSTA38 >HSP40_OSTA39 >HSP40_OSTA41 >HSP40_OSTA42 >HSP40_OSTA43 >HSP40_OSTA44

>HSP40_OSTA45 >HSP40_OSTA46 >HSP40_OSTA47 >HSP40_OSTA48 >HSP40_OSTA49 >HSP40_OSTA50

>HSP40_ARTH15 >HSP40_ARTH23 >HSP40_ARTH34 >HSP40_ARTH43 >HSP40_ARTH44 >HSP40_ARTH45

>HSP40_ARTH46 >HSP40_ARTH48 >HSP40_ARTH51 >HSP40_ARTH53 >HSP40_ARTH55 >HSP40_ARTH56

>HSP40_ARTH57 >HSP40_ARTH71 >HSP40_ARTH72 >HSP40_ARTH78 >HSP40_ARTH80 >HSP40_ARTH84

>HSP40_ARTH86 >HSP40_ARTH94 >HSP40_ARTH98 >HSP40_ARTH101>HSP40_ARTH104>HSP40_ARTH120

>HSP40_ARTH123>HSP40_ARTH127>HSP40_ARTH131>HSP40_ARTH141>HSP40_ARTH143>HSP40_ARTH147

>HSP40_ARTH148>HSP40_ARTH150>HSP40_ARTH156>HSP40_ARTH159>HSP40_ARTH160>HSP40_ARTH169

>HSP40_ARTH174>HSP40_ARTH176>HSP40_ARTH183>HSP40_ARTH184>HSP40_ARTH185>HSP40_ARTH186

>HSP40_ARTH187>HSP40_GLMA17 >HSP40_GLMA20 >HSP40_METR8 >HSP40_METR12 >HSP40_ORSA5

>HSP40_ORSA6 >HSP40_ORSA13 >HSP40_ORSA14 >HSP40_ORSA19 >HSP40_ORSA25 >HSP40_ORSA26

>HSP40_ORSA27 >HSP40_ORSA28 >HSP40_ORSA34 >HSP40_ORSA40 >HSP40_ORSA42 >HSP40_ORSA46

>HSP40_ORSA47 >HSP40_ORSA48 >HSP40_ORSA52 >HSP40_ORSA58 >HSP40_ORSA65 >HSP40_ORSA67

>HSP40_ORSA74 >HSP40_ORSA75 >HSP40_ORSA77 >HSP40_ORSA78 >HSP40_ORSA79 >HSP40_ORSA81

>HSP40_ORSA85 >HSP40_ORSA87 >HSP40_ORSA88 >HSP40_ORSA90 >HSP40_ORSA93 >HSP40_ORSA94

>HSP40_ORSA96 >HSP40_ORSA99 >HSP40_ORSA104>HSP40_ORSA107>HSP40_ORSA108>HSP40_PHPA18

>HSP40_PHPA19 >HSP40_PHPA27 >HSP40_PHPA28 >HSP40_PHPA29 >HSP40_PHPA30 >HSP40_PHPA31

>HSP40_PHPA37 >HSP40_PHPA38 >HSP40_PHPA39 >HSP40_PHPA46 >HSP40_PHPA49 >HSP40_PHPA53

>HSP40_PHPA54 >HSP40_PHPA60 >HSP40_PHPA62 >HSP40_PHPA63 >HSP40_PHPA67 >HSP40_PHPA68

>HSP40_PHPA70 >HSP40_PHPA72 >HSP40_PHPA74 >HSP40_PHPA77 >HSP40_PHPA79 >HSP40_PHPA80

>HSP40_PHPA82 >HSP40_PHPA86 >HSP40_PHPA87 >HSP40_PHPA89 >HSP40_PHPA90 >HSP40_RICO2

>HSP40_RICO19 >HSP40_RICO24 >HSP40_RICO25 >HSP40_RICO26 >HSP40_RICO27 >HSP40_RICO31

>HSP40_RICO35 >HSP40_RICO37 >HSP40_RICO39 >HSP40_RICO45 >HSP40_RICO52 >HSP40_RICO55

>HSP40_RICO61 >HSP40_RICO64 >HSP40_RICO68 >HSP40_RICO70 >HSP40_RICO76 >HSP40_RICO79

>HSP40_RICO81 >HSP40_RICO83 >HSP40_RICO88 >HSP40_RICO89 >HSP40_RICO90 >HSP40_RICO91

>HSP40_RICO92 >HSP40_RICO101>HSP40_RICO102>HSP40_SOBI1 >HSP40_SOBI2 >HSP40_SOBI21

>HSP40_SOBI25 >HSP40_SOBI29 >HSP40_SOBI31 >HSP40_SOBI33 >HSP40_SOBI35 >HSP40_SOBI36

>HSP40_SOBI40 >HSP40_SOBI41 >HSP40_SOBI43 >HSP40_SOBI44 >HSP40_SOBI47 >HSP40_SOBI48

>HSP40_SOBI58 >HSP40_SOBI62 >HSP40_SOBI64 >HSP40_SOBI72 >HSP40_SOBI75 >HSP40_SOBI77

>HSP40_SOBI80 >HSP40_SOBI83 >HSP40_SOBI85 >HSP40_SOBI86 >HSP40_SOBI87 >HSP40_SOBI88

>HSP40_SOBI90 >HSP40_SOBI93 >HSP40_SOBI101>HSP40_SOBI102>HSP40_SOBI103>HSP40_SOBI104

>HSP40_SOBI105>HSP40_SOBI107>HSP40_VIVI1 >HSP40_VIVI2 >HSP40_VIVI3 >HSP40_VIVI14

>HSP40_VIVI19 >HSP40_VIVI20 >HSP40_VIVI23 >HSP40_VIVI25 >HSP40_VIVI30 >HSP40_VIVI33

>HSP40_VIVI35 >HSP40_VIVI37 >HSP40_VIVI42 >HSP40_VIVI43 >HSP40_VIVI46 >HSP40_VIVI47

>HSP40_VIVI48 >HSP40_VIVI60 >HSP40_VIVI62 >HSP40_VIVI63 >HSP40_VIVI66 >HSP40_VIVI67

>HSP40_VIVI68 >HSP40_VIVI71 >HSP40_VIVI72 >HSP40_VIVI77 >HSP40_VIVI78 >HSP40_VIVI80

>HSP40_VIVI82 >HSP40_VIVI83 >HSP40_VIVI85 >HSP40_VIVI88 >HSP40_VIVI90 >HSP40_VIVI91

>HSP40_VIVI93 >HSP40_VIVI94 >HSP40_VIVI95 >HSP40_VIVI97 >HSP40_VIVI98 >HSP40_VIVI102

>HSP40_VIVI103>HSP40_ZEMA11 >HSP40_ZEMA49 >HSP40_ZEMA50 >HSP40_ZEMA72 >HSP40_ZEMA81

>HSP40_ZEMA82 >HSP40_ZEMA87 >HSP40_AEAE10 >HSP40_AEAE16 >HSP40_AEAE22 >HSP40_AEAE25

>HSP40_AEAE26 >HSP40_ANGA6 >HSP40_ANGA8 >HSP40_ANGA9 >HSP40_ANGA11 >HSP40_ANGA13

>HSP40_ANGA16 >HSP40_ANGA21 >HSP40_ANGA25 >HSP40_ANGA26 >HSP40_ANGA29 >HSP40_ANGA31

>HSP40_ANGA33 >HSP40_ANGA34 >HSP40_ANGA35 >HSP40_ANGA36 >HSP40_BOMO6 >HSP40_BOMO8

>HSP40_BOMO11 >HSP40_BRMA3 >HSP40_BRMA6 >HSP40_BRMA8 >HSP40_BRMA12 >HSP40_BRMA13

>HSP40_BRMA14 >HSP40_BRMA16 >HSP40_BRMA17 >HSP40_BRMA18 >HSP40_BRMA19 >HSP40_BRMA21

>HSP40_BRMA23 >HSP40_BRMA24 >HSP40_BRMA25 >HSP40_BRMA26 >HSP40_BRMA27 >HSP40_BRMA28

>HSP40_BOTA35 >HSP40_BOTA37 >HSP40_BOTA44 >HSP40_BOTA46 >HSP40_BOTA54 >HSP40_CABR3

>HSP40_CABR6 >HSP40_CABR9 >HSP40_CABR10 >HSP40_CABR15 >HSP40_CABR17 >HSP40_CABR18

>HSP40_CABR22 >HSP40_CABR23 >HSP40_CABR24 >HSP40_CABR25 >HSP40_CABR26 >HSP40_CABR29

>HSP40_CABR31 >HSP40_CABR32 >HSP40_CAEL4 >HSP40_CAEL6 >HSP40_CAEL13 >HSP40_CAEL15

>HSP40_CAEL17 >HSP40_CAEL18 >HSP40_CAEL25 >HSP40_CAEL27 >HSP40_CAEL36 >HSP40_CIIN3

>HSP40_CIIN7 >HSP40_CIIN9 >HSP40_CIIN10 >HSP40_CIIN12 >HSP40_CIIN13 >HSP40_CIIN14

>HSP40_CIIN15 >HSP40_CIIN16 >HSP40_CIIN17 >HSP40_CIIN18 >HSP40_CIIN20 >HSP40_CIIN22

>HSP40_CIIN23 >HSP40_CIIN25 >HSP40_CIIN26 >HSP40_CIIN28 >HSP40_CIIN33 >HSP40_DARE12

>HSP40_DARE17 >HSP40_DARE29 >HSP40_DARE35 >HSP40_DARE37 >HSP40_DARE40 >HSP40_DARE59

>HSP40_DAPU5 >HSP40_DAPU9 >HSP40_DAPU11 >HSP40_DAPU12 >HSP40_DAPU16 >HSP40_DAPU22

>HSP40_DAPU24 >HSP40_DAPU25 >HSP40_DAPU31 >HSP40_DAPU33 >HSP40_DRME3 >HSP40_DRME8

>HSP40_DRME9 >HSP40_DRME12 >HSP40_DRME16 >HSP40_DRME18 >HSP40_DRME19 >HSP40_DRME23

>HSP40_DRME24 >HSP40_DRME25 >HSP40_DRME27 >HSP40_DRME33 >HSP40_DRME36 >HSP40_DRME42

>HSP40_DRME43 >HSP40_DRME45 >HSP40_DRME47 >HSP40_DRME48 >HSP40_DRME52 >HSP40_DRME53

>HSP40_DRME56 >HSP40_GAGA25 >HSP40_HOSA14 >HSP40_HOSA32 >HSP40_HOSA36 >HSP40_HOSA85

>HSP40_HOSA87 >HSP40_HOSA107>HSP40_HOSA117>HSP40_MAMU7 >HSP40_MAMU14 >HSP40_MAMU25

>HSP40_MAMU44 >HSP40_MODO7 >HSP40_MODO20 >HSP40_MODO25 >HSP40_MODO26 >HSP40_MODO38

>HSP40_MODO39 >HSP40_MODO45 >HSP40_MUMU16 >HSP40_MUMU85 >HSP40_MUMU91 >HSP40_MUMU92

>HSP40_MUMU93 >HSP40_MUMU105>HSP40_MUMU108>HSP40_MUMU118>HSP40_MUMU138>HSP40_PATR30

>HSP40_RANO9 >HSP40_RANO38 >HSP40_RANO77 >HSP40_RANO87 >HSP40_SCJP7 >HSP40_SCJP8

>HSP40_SCJP11 >HSP40_SCJP13 >HSP40_SCJP14 >HSP40_SCJP15 >HSP40_SCJP16 >HSP40_SCJP17

>HSP40_SCJP18 >HSP40_SCJP19 >HSP40_SCJP21 >HSP40_SCJP23 >HSP40_SCJP24 >HSP40_SCJP26

>HSP40_SCJP27 >HSP40_SCJP33 >HSP40_SCJP35 >HSP40_SCJP37 >HSP40_TENI1 >HSP40_TENI3

>HSP40_TENI6 >HSP40_TENI11 >HSP40_TENI12 >HSP40_TENI13 >HSP40_TENI17 >HSP40_TENI20

>HSP40_TENI24 >HSP40_TENI25 >HSP40_TENI26 >HSP40_TENI28 >HSP40_TENI29 >HSP40_TENI35

>HSP40_TENI40 >HSP40_TENI42 >HSP40_TENI46 >HSP40_TRCA5 >HSP40_TRCA10 >HSP40_TRCA11

>HSP40_TRCA13 >HSP40_TRCA18 >HSP40_TRCA30 >HSP40_TRCA32 >HSP40_TRCA33 >HSP40_TRCA36

>HSP40_TRCA37 >HSP40_TRCA38 >HSP40_TRCA39 >HSP40_XETR26 >HSP40_XETR34 >HSP40_DIFA29

>HSP40_PHIN44 >HSP40_PLFA45 >HSP40_TETH28 >HSP40_TRVA44 >HSP40_CATR25 >HSP40_CRNE25

>HSP40_PYTR33 >HSP40_SCPO26 >HSP40_YALI25 >HSP40_CHRE63 >HSP40_MIPU67 >HSP40_OSTA54

>HSP40_ORSA109>HSP40_ORSA110>HSP40_VIVI105>HSP40_ANGA37 >HSP40_BRMA31 >HSP40_DRME58

>HSP40_MODO47

# HSP60(163)

>GroES_ACMA2 >GroES_ACLA1 >GroES_BDBA2 >GroEL_BEIN2 >THS_CAHY1 >GroEL_CHTR2

>GroEL_CHTR3 >GroES_CHTR1 >GroEL_CHAB2 >GroES_HEPY1 >GroEL_MECA3 >GroEL_MILU3

>GroES_MYPN1 >GroES_SAPA1 >GroES_STTH1 >GroES_STGR2 >GroES_VIVU3 >GroES_XAOR1

>THSB_HASP1 >THS_HAMU4 >THS_HALA1 >THSB_HYBU1 >GroES_MEAC1 >THS_MEAC4

>THS_MEMA1 >CPN60_CRHO1 >CCT3_CRPR1 >CCT8_CRPR1 >CPN10_CRPR1 >CPN60_DIDI2

>CPN60_DIDI3 >CPN60_DIDI4 >CPN60_DIDI5 >CPN60_DIDI6 >CPN60_DIDI7 >CPN60_DIDI8

>CPN60_DIDI9 >FAB1_DIDI1 >CPN10_DIDI1 >CCT6_ENHI2 >CCT7_ENHI2 >CCT8_ENHI2

>CPN10_ENHI1 >CCT3_GIIN1 >CCT7_GIIN1 >CPN60_GILA1 >CCT8_GILA1 >FAB1_PHIN1

>FAB1_PHIN2 >CPN60_PHIN2 >CPN60_PHIN3 >CPN60_PHIN5 >CCT6_PHIN1 >CPN60_PLFA2

>FAB1_PLVI1 >CCT8_PLVI1 >CPN10_PLVI2 >CPN60_TETH2 >FAB1_TETH1 >CCT4_TETH1

>CCT6_TRVA2 >CCT7_TRVA1 >CCT8_TRVA2 >CPN10_TRVA1 >CPN10_TRVA2 >CPN10_ASCL1

>CCT5_ASFL1 >CCT8_ASNI1 >CPN10_ASOR1 >CCT1_ASTE1 >FAB1_CAAL1 >FAB1_CATR1

>FAB1_CRNE1 >FAB1_DEHA1 >CPN60_KLLA2 >FAB1_KLLA1 >CCT2_MICA1 >CCT4_MICA2

>FAB1_NEFI1 >FAB1_NECR1 >FAB1_PIST1 >CCT8_PYTR1 >FAB1_PYTR1 >FAB1_SACE1

>FAB1_SCJA1 >FAB1_SCPO1 >FAB1_YALI1 >FAB1_CHRE1 >CPN10_CHRE2 >CPN10_CHRE3

>CPN10_CHRE4 >CPN60_MIPU4 >CPN10_MIPU2 >CPN10_MIPU3 >CPN10_OSLU1 >CPN60_OSTA3

>CPN10_OSTA1 >CPN10_OSTA3 >CPN60_ARTH6 >CCT5_ARTH3 >FAB1_ARTH1 >FAB1_ARTH2

>FAB1_ARTH3 >FAB1_ARTH5 >CPN10_ARTH4 >CPN60_ORSA10 >CCT2_ORSA2 >CCT3_ORSA3

>CCT4_ORSA1 >FAB1_ORSA1 >FAB1_ORSA5 >FAB1_ORSA6 >CPN10_ORSA1 >CPN10_ORSA4

>FAB1_PHPA2 >FAB1_PHPA3 >CPN10_PHPA9 >FAB1_RICO3 >FAB1_RICO4 >FAB1_SOBI4

>FAB1_SOBI7 >FAB1_SOBI8 >CPN60_VIVI10 >FAB1_VIVI1 >FAB1_VIVI2 >FAB1_VIVI6

>CPN60_VIVI12 >CPN60_VIVI13 >CPN10_ZEMA14 >CPN10_ZEMA16 >CPN10_ZEMA18 >FAB1_AEAE1

>FAB1_ANGA1 >CPN60_BOTA4 >FAB1_BRMA1 >CCT8_CABR1 >FAB1_CAEL1 >CPN60_CALU3

>FAB1_CIIN1 >CPN60_DARE3 >CPN60_DARE5 >CCT7_DARE2 >FAB1_DARE1 >CPN60_DAPU2

>CPN60_DRME7 >FAB1_DRME2 >CPN60_HOSA11 >CPN60_HOSA12 >CCT8_HOSA6 >CPN10_HOSA7

>CPN60_MODO5 >CPN60_MUMU12 >FAB1_MUMU3 >CPN60_TENI1 >CPN60_TENI2 >CCT1_TENI1

>CCT3_TENI1 >FAB1_TENI1 >CPN10_TENI1 >FAB1_TRCA1 >CPN10_TRCA2 >CPN60_XETR3

>CPN60_XETR4

# HSP70(283)

>HSP70_ACMA4 >HSP70_AGVI3 >HSP70_ANVA5 >HSP70_BAAN2 >HSP70_BACE2 >HSP70_BAFR2

>HSP70_BDBA2 >HSP70_BOBU2 >HSP70_CACR2 >HSP70_CLKL4 >HSP70_CLKL5 >HSP70_CLKL8

>HSP70_CLKL9 >HSP70_CLPE2 >HSP70_CLTE2 >HSP70_COEF2 >HSP70_DEAU2 >HSP70_DEAU4

>HSP70_DEMA2 >HSP70_LIMO2 >HSP70_MEFL3 >HSP70_MECA3 >HSP70_MECA4 >HSP70_MIAE1

>HSP70_MYAV4 >HSP70_MYBO3 >HSP70_MYSM4 >HSP70_MYSM5 >HSP70_MYUL2 >HSP70_MYXA5

>HSP70_MYXA6 >HSP70_MYXA8 >HSP70_MYXA10 >HSP70_MYXA11 >HSP70_MYXA15 >HSP70_NEGO2

>HSP70_NOFA2 >HSP70_NOFA3 >HSP70_NOFA4 >HSP70_NOFA5 >HSP70_NOFA6 >HSP70_PHPR6

>HSP70_PRAC2 >HSP70_PSIN4 >HSP70_PSIN6 >HSP70_RHSP2 >HSP70_RIRI2 >HSP70_STGR2

>HSP70_VIHA4 >HSP70_VISP3 >HSP70_VIVU4 >HSP70_XAOR2 >HSP70_HAMU2 >HSP70_MEHU2

>HSP70_CRHO3 >HSP70_DIDI3 >HSP70_DIDI6 >HSP70_ENHI2 >HSP70_ENHI3 >HSP70_ENHI4

>HSP70_ENHI5 >HSP70_ENHI6 >HSP70_ENHI7 >HSP70_ENHI11 >HSP70_ENHI14 >HSP70_ENHI20

>HSP70_ENHI21 >HSP70_ENHI22 >HSP70_ENHI23 >HSP70_ENHI26 >HSP70_ENHI31 >HSP70_ENHI32

>HSP70_ENHI35 >HSP70_ENHI42 >HSP70_ENHI48 >HSP70_ENHI55 >HSP70_ENHI56 >HSP70_GIIN3

>HSP70_GIIN5 >HSP70_GILA5 >HSP70_GILA6 >HSP70_LEBR1 >HSP70_LEBR6 >HSP70_LEBR7

>HSP70_LEBR9 >HSP70_LEBR11 >HSP70_LEBR12 >HSP70_LEBR13 >HSP70_PHIN9 >HSP70_PHIN10

>HSP70_PHIN11 >HSP70_PHIN12 >HSP70_PHIN13 >HSP70_PHIN17 >HSP70_PHIN18 >HSP70_PHIN19

>HSP70_PLFA4 >HSP70_PLVI4 >HSP70_TETH6 >HSP70_TETH7 >HSP70_TETH8 >HSP70_TETH13

>HSP70_TETH14 >HSP70_TETH15 >HSP70_TETH16 >HSP70_TRVA4 >HSP70_TRVA10 >HSP70_TRVA21

>HSP70_TRVA22 >HSP70_TRVA23 >HSP70_TRVA25 >HSP70_TRVA27 >HSP70_TRVA28 >HSP70_TRVA29

>HSP70_TRVA30 >HSP70_TRVA39 >HSP70_TRVA40 >HSP70_TRVA41 >HSP70_TRVA43 >HSP70_TRVA44

>HSP70_TRVA45 >HSP70_TRVA46 >HSP70_TRVA48 >HSP70_TRVA50 >HSP70_TRVA51 >HSP70_TRVA52

>HSP70_TRVA55 >HSP70_TRVA56 >HSP70_TRVA57 >HSP70_TRVA58 >HSP70_TRVA59 >HSP70_TRVA60

>HSP70_TRVA61 >HSP70_TRVA62 >HSP70_TRVA63 >HSP70_TRVA66 >HSP70_TRVA67 >HSP70_TRVA68

>HSP70_TRVA69 >HSP70_TRVA71 >HSP70_TRVA72 >HSP70_TRVA73 >HSP70_TRVA74 >HSP70_TRVA75

>HSP70_TRVA79 >HSP70_TRVA80 >HSP70_TRVA81 >HSP70_TRVA82 >HSP70_TRVA83 >HSP70_TRVA84

>HSP70_TRVA85 >HSP70_TRVA86 >HSP70_TRVA88 >HSP70_TRVA89 >HSP70_TRVA92 >HSP70_TRVA93

>HSP70_TRVA94 >HSP70_TRVA95 >HSP70_TRVA96 >HSP70_TRVA97 >HSP70_ASGO5 >HSP70_ASCL9

>HSP70_ASFL2 >HSP70_ASFL4 >HSP70_ASFL5 >HSP70_ASFL13 >HSP70_ASFU6 >HSP70_ASFU11

>HSP70_ASNI1 >HSP70_ASNI3 >HSP70_ASNI12 >HSP70_ASNG1 >HSP70_ASNG5 >HSP70_ASNG6

>HSP70_ASNG8 >HSP70_ASNG12 >HSP70_ASNG13 >HSP70_ASNG14 >HSP70_ASNG15 >HSP70_ASNG16

>HSP70_ASOR11 >HSP70_ASTE4 >HSP70_ASTE10 >HSP70_ASTE11 >HSP70_CATR3 >HSP70_CRNE3

>HSP70_CRNE5 >HSP70_CRNE8 >HSP70_KLLA7 >HSP70_NEFI6 >HSP70_NEFI9 >HSP70_NEFI13

>HSP70_NEFI14 >HSP70_NECR4 >HSP70_NECR7 >HSP70_NECR11 >HSP70_NECR12 >HSP70_PECH4

>HSP70_PIPA7 >HSP70_PIST2 >HSP70_PIST3 >HSP70_PIST11 >HSP70_PYTR8 >HSP70_PYTR9

>HSP70_YALI1 >HSP70_CHRE2 >HSP70_CHRE4 >HSP70_CHRE9 >HSP70_CHRE12 >HSP70_CHRE15

>HSP70_MIPU2 >HSP70_MIPU4 >HSP70_MIPU8 >HSP70_MIPU9 >HSP70_MIPU10 >HSP70_OSLU2

>HSP70_OSTA2 >HSP70_ARTH4 >HSP70_METR4 >HSP70_ORSA13 >HSP70_ORSA17 >HSP70_ORSA18

>HSP70_ORSA24 >HSP70_ORSA27 >HSP70_ORSA28 >HSP70_ORSA29 >HSP70_ORSA33 >HSP70_ORSA34

>HSP70_ORSA37 >HSP70_PHPA5 >HSP70_PHPA28 >HSP70_PHPA29 >HSP70_PHPA30 >HSP70_PHPA32

>HSP70_RICO12 >HSP70_RICO13 >HSP70_RICO14 >HSP70_SOBI4 >HSP70_SOBI5 >HSP70_SOBI15

>HSP70_SOBI26 >HSP70_SOBI28 >HSP70_SOBI35 >HSP70_SOBI36 >HSP70_TRAE5 >HSP70_VIVI1

>HSP70_VIVI2 >HSP70_VIVI3 >HSP70_VIVI8 >HSP70_VIVI9 >HSP70_VIVI13 >HSP70_VIVI16

>HSP70_VIVI19 >HSP70_VIVI22 >HSP70_VIVI25 >HSP70_VIVI27 >HSP70_VIVI28 >HSP70_ZEMA36

>HSP70_ANGA5 >HSP70_ANGA7 >HSP70_BRMA5 >HSP70_BRMA6 >HSP70_BRMA9 >HSP70_CABR2

>HSP70_CABR5 >HSP70_CABR8 >HSP70_CAEL2 >HSP70_CAEL15 >HSP70_CIIN7 >HSP70_DARE32

>HSP70_DRME41 >HSP70_HOSA47 >HSP70_MUMU42 >HSP70_SCJP6 >HSP70_SCJP8 >HSP70_SCJP9

>HSP70_TENI8 >HSP70_TENI10 >HSP70_TENI11 >HSP70_TENI13 >HSP70_TRCA8 >HSP70_TRCA12

>HSP70_TRCA15

# HSP90(58)

>HTPG_ACMA1 >HTPG_BAAM1 >HTPG_BAFR1 >HTPG_BAFR2 >HTPG_BEIN1 >HTPG_BOBU1

>HTPG_CLDI1 >HTPG_COGL1 >HTPG_DEDE1 >HTPG_LIMO1 >HTPG_MYLE1 >HTPG_MYXA1

>HTPG_MYXA2 >HTPG_RIRI1 >HTPG_XACA2 >HSP90_CRHO2 >TRAP1_DIDI1 >GRP94_GILA1

>GRP94_LEBR1 >GRP94_PHIN1 >TRAP1_PLFA1 >GRP94_PLFA1 >GRP94_PLVI2 >TRAP1_TETH1

>HSP90_TRVA2 >GRP94_CRNE1 >TRAP1_MIPU1 >GRP94_MIPU1 >TRAP1_OSTA1 >HSP90_ORSA4

>GRP94_PHPA3 >GRP94_PHPA4 >HSP90_VIVI5 >HSP90_VIVI8 >HSP90_VIVI9 >HSP90_VIVI10

>HSP90_VIVI11 >HSP90_VIVI12 >HSP90_VIVI14 >TRAP1_VIVI2 >GRP94_VIVI2 >HSP90C_VIVI2

>HSP90_BOTA6 >HSP90_CALU20 >TRAP1_CIIN1 >GRP94_DRME2 >HSP90_HOSA8 >HSP90_HOSA14

>HSP90_HOSA16 >HSP90_HOSA17 >HSP90_HOSA30 >GRP94_HOSA6 >GRP94_MAMU2 >TRAP1_MUMU7

>HSP90_RANO10 >GRP94_SCJP1 >HSP90_TENI3 >TRAP1_TRCA1

# HSP100(85)

>HslU_ACCA1 >ClpA_AGRA1 >ClpA_BAFR1 >ClpX_BIAD1 >ClpC_BIAN2 >ClpC_BOBU1

>ClpA_BOBU1 >ClpA_CAFE1 >ClpA_CAHO1 >ClpC_DERA1 >ClpX_DEAU2 >ClpX_DEMA2

>ClpA_FLPS1 >ClpA_HEPY1 >HSP100_LEME1 >HSP100_LEME3 >HSP100_LEME4 >ClpB_MELO2

>ClpB_MEFL1 >ClpB_MENO3 >ClpA_MIAE1 >ClpB_MYXA1 >ClpB_MYXA4 >ClpA_MYXA2

>ClpA_MYXA3 >ClpA_MYXA4 >ClpX_MYXA3 >ClpX_MYXA4 >ClpB_PHPR2 >ClpB_PRMI2

>ClpA_PSAE2 >ClpB_PSPU3 >ClpC_STTH1 >ClpA_TRPA1 >ClpB_XAOR3 >ClpB_YEPE2

>ClpB_YEPE3 >ClpA_MEHU1 >ClpA_METE1 >ClpA_CRHO1 >HslU_DIDI1 >HslU_LEBR2

>ClpX_PHIN2 >HslU_PHIN1 >HSP101_PLFA1 >ClpB_PLFA1 >ClpB_PLFA2 >HslU_PLFA1

>ClpB_PLVI2 >HSP104_ASFU1 >ClpX_ASFU1 >ClpX_PECH1 >ClpX_PIPA1 >HSP100_PIST1

>ClpX_SACE1 >ClpX_YALI1 >ClpB_CHRE2 >ClpB_CHRE4 >ClpX_CHRE1 >HSP100_CHRE1

>ClpB_MIPU2 >ClpC_MIPU2 >ClpX_OSLU1 >ClpB_OSTA1 >ClpB_OSTA3 >HslU_OSTA1

>ClpC_ARTH2 >HSP100_ARTH2 >ClpB_ORSA1 >ClpB_ORSA3 >ClpC_ORSA2 >HslU_PHPA1

>HSP100_RICO2 >ClpX_RICO4 >ClpA_SOBI1 >ClpA_SOBI2 >ClpX_SOBI3 >HSP100_VIVI2

>ClpX_VIVI1 >ClpX_ANGA1 >ClpX_BRMA2 >ClpX_CABR1 >ClpX_CAEL3 >ClpB_HOSA1

>ClpX_TENI1
