# Supplementary material for: Identifying Heat Shock Protein Families from Imbalanced Data by Using Combined Features
Source: Comput Math Methods Med. 2020 Sep 23;2020:8894478. doi: 10.1155/2020/8894478 (PMC7530508; doi:10.1155/2020/8894478)
Supplement: Supplementary 2 — The sequence names of the independent datasets. [file 8894478.f2.docx]

The independent datasets including two datasets: HGNC Dataset and RICE dataset

**HGNC(96)**

>P04792 >Q16082 >Q12988 >P02489 >P02511 >O14558 >Q9UBY9 >Q9UJY1 >Q9BQS6 >Q14990

>Q9Y547 >P31689 >O60884 >Q96EY1 >Q8WW22 >P25685 >P25686 >Q8WWF6 >Q9UDY4 >O75953

>O75190 >Q7Z6W7 >Q8NHS0 >Q9UBS3 >Q9UBS4 >Q9NXW2 >P59910 >Q8TBM8 >Q96KC8 >Q99543

>Q13217 >Q9NNZ3 >Q9H3Z4 >Q9UF47 >Q8N7S2 >O75061 >Q99615 >O75937 >Q8WXX5 >Q8IXB1

>Q9NVH1 >Q9UKB3 >O75165 >Q6Y2X3 >Q9Y5T4 >Q9Y2G8 >Q9NVM6 >Q9H819 >Q96DA6 >Q8IWL3

>Q5F1R6 >Q8N4W6 >Q9UGP8 >Q6P3W2 >Q9H1X3 >O14976 >Q9NZQ0 >Q9NX36 >Q9NZJ4 >Q96LL9

>Q8TAM1 >Q6ZW61 >P17987 >P78371 >P49368 >P50991 >P48643 >P40227 >Q92526 >Q99832

>P50990 >Q9H078 >P10809 >P61604 >Q9NPJ1 >P0DMV8 >P0DMV9 >P34931 >P54652 >P34932

>O95757 >P11021 >P17066 >P48741 >P11142 >P38646 >O43301 >Q96MM6 >P48723 >Q0VDF9

>Q92598 >Q9Y4L1 >P07900 >P08238 >P14625 >Q12931

**RICE(55)**

**Wang et al.(31)**

>Q6K7E9.1 >Q84J50.1 >P27777.1 >Q0E4A8.1 >Q67X83.1 >B7EZJ7.1 >Q6Z7V2.1

>Q84Q72.1 >Q84Q77.1 >P31673.2 >Q7EZ57.1 >P0C031.2 >Q943E9.1 >Q6AUW3.1

>Q6Z7B0.1 >Q0J4P2.2 >Q69QQ6.1 >Q75GT3.1 >Q0E3C8.3 >Q6F2Y7.1 >Q10SR3

>Q10PW8 >Q9AQZ5 >Q2QV45 >Q943K7 >Q10NA9 >Q5Z9N8 >Q8H903

>Q10RW9 >Q9LWT6

>Q7X9A7

**Sarkar et al.(24)**

>LOC_Os01g62290.1 >LOC_Os03g16860.1 >LOC_Os03g16880.1 >LOC_Os03g16920.1

>LOC_Os03g60620.1 >LOC_Os05g38530.1 >LOC_Os11g47760.1 >LOC_Os11g08440.1

>LOC_Os11g08445.1 >LOC_Os11g08460.1 >LOC_Os11g08470.1 >LOC_Os12g38180.1

>LOC_Os02g02410.1 >LOC_Os03g50250.1 >LOC_Os05g30480.1 >LOC_Os05g35400.1

>LOC_Os08g09770.1 >LOC_Os01g33360.1 >LOC_Os02g53420.1 >LOC_Os03g02260.1

>LOC_Os09g31486.1 >LOC_Os05g23740.1 >LOC_Os12g14070.1 >LOC_Os01g49430.1
